# Cincinnati Academy of Medicine

**Published:** 1881-03

**Authors:** 


### Reports of Societies.

---

### CINCINNATI ACADEMY OF MEDICINE.

*Fracture of the Skull.*—Dr. Luff reported the following case :

On Saturday, Jan. 29th, at 6 o'clock, p.m., he was called to attend Charles McDowell, a young man, aged 19, whom he found in a comatose condition, breathing stertorious, drawing long sighs at intervals, intermittent convulsive action of both arms and hands with a slight tendency to opisthotonus; temperature of the axilla normal, pulse full and decided but very slow; some œdema of the left temple, but no perceptible wound or brasion of the skin; muscles of the jaw rigid, mouth clinched, mucus rales in the throat; right eye normal, left pupil highly dilated. No fracture or depression at the seat of injury could be discovered.

On interrogation the speaker was informed that the patient had been struck with a club at 5 p.m.; that he had ridden on a locomotive to his aunt's residence—a distance of nearly two miles—where he jumped off and told his relatives a boy had struck him. These were the last words he uttered. He immediately fell into a comatose condition, in which he remained until his death, which occurred at 1 a.m., the next day.

A post-mortem examination held in the afternoon revealed the following: Considerable quantity of blood beneath the scalp, left temporal muscle highly engorged, below this a fracture extending upward and backward four inches and a half, transversely one inch, blood still exuding through the line of fracture. After removing the skull cap a blood-clot estimated at four ounces was discovered directly at the seat of fracture. The convexity of the brain was destroyed and this organ very much flattened and engorged with blood on its superfices. The fracture continued inwards to the middle line of the sphenoid bone, but was attented with no depression or spicula of bone pointing inwards—a simple fracture, accompanied with rupture, of the meningeal artery and subsequent consequences. All other organs were found in a perfectly healthy condition.

In view of the fact that compression takes place from ruptured vessels—at times upon the side opposite the seat of injury—and when the trephine is used at the seat of injury fatal hemorrhage has occurred, and also that patients sometimes recover even where evidence of compression is shown, the question arises whether it would have been proper to use the trephine in this instance.

*Discussion.*—Dr. Whittaker said that although these cases belonged purely to the domain of surgery every physician was interested in the general subject of hemorrhage into the brain. We see cases of apoplexy from cerebral hemorrhage presenting at times the very same train of symptoms shown in this case. For the most part, however, a patient falls over suddenly in apoplexy, which is, indeed, the meaning of the word, though an absolutely sudden death from apoplexy is very rare and means always hemorrhage into the medulla.

The classical centre of spontaneous extravasation is about the optostriatic bodies, and Charcot, Bouchard and Zenker have all shown that all cases, almost without exception, depend upon rupture of miliary aneurisms, for the most part of minute, if not of microscopic size. The most interesting feature connected with these hemorrhages concerns their localization. In a case like the one reported, the trauma might indicate the seat of the lesion, but in spontaneous rupture we may be guided only by the effect. Nothnagel has shown that a pretty accurate localization is possible after the lapse of six hours, by which time all symptoms of pressure or reflected irritation, the so-called indirect symptoms, have subsided. Hemmorrhage into the cerebellum, at least into the vermiform process of it, gives cerebella ataxia; into the medulla, if not quickly fatal, to disturbance of respiration and circulation, difficulty of speech and deglutition and diabetes; into the crus cerebelli to compulsory movements and deviation of the planes of the eyes; into the pons, that is, in its lower half, to crossed paralysis of the face and body; into the crus cerebri to paralysis of the occulo-motor nerve with crossed facial or corporal paralysis; into the corpus straitum to temporary or permanent hemiphlegia, according as the anterior portion of the internal capsule is broken up or not, while isolated paralyses or monoplegiæ, or isolated spasms indicate cortical lesions.

Dr. Buckner, in answer to a question, said that he knew nothing besides compression that would as rapidly produce dilatation of the pupil, causing a paralysis of those branches of the third nerve which supply the circular muscle of the iris. He would, however, like to know if it would be justifiable to trephine if no depression could be felt, but only symptoms of compression were manifest.

Dr. Conner remarked that the case reported was a typical one. Of special diagnostic value was the dilated condition of the left pupil, which indicated pressure in the sphenoidal fissure, so complete as to destroy the action of the third nerve. There was evidently a rupture of the middle meningeal artery. The primary unconsciousness resulting from the hemorrhage is usually of short duration; the patient may not regain his senses until the second or third, sometimes even as late as the sixth or eighth day, when another attack of unconsciousness comes on, from which he never rallies. Besides flattening of the surface, as in this case, a cup-shaped depression may at times occur in the brain. The speaker remembered one instance in which he removed a clot large enough to fill both hands. A favorable termination may be hoped for only when one of two things takes place: either the clot being absorbed or becoming encysted—forming an arachnoidal cyst.

The question had been asked whether an operation would be justifiable. He would answer, that if consent for that purpose could be obtained it certainly would be, as it then afforded a chance of reaching and relieving the effusion; if, however, the extent of the effusion be very great, the prospects were less favorable. There is always a great liability to new hemorrhage, which must be carefully guarded against. After pus has once formed, the outlook for relief by operative measures is decidedly unfavorable. In London not a single case where the trephine has been used for pus has terminated favorably. This is somewhat startling, since although such is the rule, a few cases of recovery have been elsewhere recorded.

In conclusion, the speaker would caution against the indiscriminate use of styptics, especially presulphate of iron, in these cases. A man was brought to the Cincinnati Hospital the week previous, who had received an injury on the right side of his head, the laceration being of horse-shoe shape and measuring 3½ to 4 inches in diameter. Before his admission to the hospital a large amount of presulphate of iron had been put in the wound to arrest the hemorrhage. The hair was thoroughly matted with the salt, and the parts underneath emitted a horrible stench emanating from a large mass of decomposed blood and pus. It took a long time to remove this coating, and the head had to be shaved clean. Hemorrhage proceeding from the posterior branch of the temporal artery was readily stopped by securing this vessel; but two inches of the skull were already in a state of necrosis and denuded of periosteum, which latter were gangrenous. Had sutures been placed in the wound and compression made, it would have been far better than to resort to this abuse of the styptic, whereby all attempts at union by first intention had been frustrated and recovery unnecessarily delayed for a long time.—*Cincinnati Lancet and Clinic.*

